# Designing Optimal Food-Based Recommendations and Nutrient-Dense Canteen Menu for Oil and Gas Workers Using Linear Programming: A Preliminary Study in Oil and Gas Worksite in East Kalimantan, Indonesia

**DOI:** 10.3390/nu15194132

**Published:** 2023-09-25

**Authors:** Nur Lailatuz Zahra, Dian Novita Chandra, Muchtaruddin Mansyur, Umi Fahmida

**Affiliations:** 1 Southeast Asian Ministers of Education Organization Regional Centre for Food and Nutrition (SEAMEO RECFON), Jakarta 13120, Indonesia; nurlailatuzzahra@gmail.com (N.L.Z.); muchtaruddin.mansyur@ui.ac.id (M.M.); 2 Department of Nutrition, Faculty of Medicine, Universitas Indonesia—Dr. Cipto Mangunkusumo General Hospital, Jakarta 10430, Indonesia; dian.chandra@ui.ac.id; 3 Department of Community Medicine, Faculty of Medicine, Universitas Indonesia—Dr. Cipto Mangunkusumo General Hospital, Jakarta 10430, Indonesia

**Keywords:** food-based recommendation, linear programming, oil and gas worker, overweight, nutrient-dense menu, nutrient gap

## Abstract

The objective of this study is to identify problem nutrients and to develop food-based recommendations (FBRs) and nutrient-dense menus based on the nutrient gaps. This study was conducted among male workers (*n* = 31) in an oil and gas worksite in East Kalimantan, Indonesia. Body weight, height, waist circumference, as well as systolic and diastolic blood pressure were measured. Weekly food consumption patterns were assessed using 1 day 24 h dietary recall (24HR), 1 day weighed food record (WFR), and 5 day food tally. Linear programming (LP) analysis using WHO Optifood software was used to identify problem nutrients and develop FBRs. The identified nutrient gaps were inserted in the nutrient-dense menu for the worksite canteen. Obesity, central obesity, and hypertension were reported in 64.5%, 48.4%, and 3.2% of the workers. Calcium, folate, total PUFA, *n*-6 PUFA, and dietary fiber were identified as problem nutrients. The FBRs can improve the intake of problem nutrients from 20% of recommended nutrient intake (RNI) in the nonoptimized diet to 50–70% RNI in the optimized diet, while controlling the intake of sodium and saturated fat within an acceptable range. The remaining nutrient gaps (calcium, total PUFA, *n*-6 PUFA, and dietary fiber) were inserted into the 14-day modified canteen snack menu. This study provides initial evidence that a combination of FBRs and a modified canteen menu can optimize the diet of the workers. Further studies assessing the effectiveness of the developed FBRs and modified menus are needed.

## 1. Introduction

The workplace meal program is an important investment in malnutrition, obesity, and chronic disease prevention [1]. Recently, workforce nutrition has been added as one of the indicators of the 2023 Food and Agriculture Benchmark, considering that 58% of the world’s adult population spends a third of their time at work. Companies are accountable to provide nutrition intervention in the workplace to improve the awareness, access, and supply of healthy food [2]. A systematic review of the interventions study conducted in a workplace canteen and other supporting components showed a consistently higher intake of fruits and vegetables and improved dietary intake. The workplace food environment is a major determinant of workers’ dietary intake [3]. The global burden of disease attributable to dietary risk factors (led by high intake of sodium together with low intake of whole grains and fruits) was estimated to have caused approximately 11 million deaths and 255 million lost years of healthy life in 2017 [4]. Especially in the working-age population, poor diet and excess body weight have become a major concern since they directly affect work performance [5]. Particularly in oil and gas industries, 80% of obese workers were more likely to have a higher number of absence days compared to normal-weight employees. This was attributable to cardiovascular diseases (CVDs) illness with around USD 1.8 million of economic loss [6].

Compared to general workers, there is an increased risk for obesity, high blood pressure, high fasting blood glucose, and high cholesterol level among workers in oil and gas settings [7,8], due to (1) long working hours (12 h long shifts with 12–14 days rotation) that may lead to poor dietary habits, eating and snacking frequency, food choice, and energy distribution [9]; (2) exposure to the obesogenic food environment, which includes energy-dense palatable food accessible and available any time of the day, overuse of oil, salt, cheese, and sauces; large portion size and lack of healthy alternatives in canteens [10,11]; (3) insufficient physical activity, fatigue due to a demanding schedule and uncommon working hours which may result in difficulty in engaging with social forms of physical exercise or team sports [12]. In East Kalimantan, Indonesia, the prevalence of obesity among oil and gas workers was 49.5%. More than half of the workers had a higher fat intake and only 12.4% had an adequate dietary fiber intake. The workers had difficulty in implementing and maintaining an active lifestyle; 79.1% were not exercising regularly [13,14].

Oil and gas industries invested in worksite wellness programs designed to improve employees’ overall health and well-being, including food and nutrition [10,15,16]. However, the evidence is rather limited. There is currently only one RCT with a medical care and nutrition education intervention aimed at petrochemical workers with dyslipidemia [17]. Positive aspects of the oil and gas workplace setting include supportive management, availability of occupational health personnel, modifiable food, and physical activity environment [6,18]. Companies and academics must work together to develop a worksite wellness program that is grounded in evidence to achieve good nutrition and health status of the workers.

To improve the quality of workers’ diets, linear programming (LP) can be used to find the optimal combination of daily food patterns for a specific population under various constraints. In LP, the goal is to meet energy and nutrient requirements while keeping the constraints, i.e., minimizing deviation from existing diets and (optionally) low cost [19,20]. In general, a dietary guideline that is developed based on existing food patterns is more feasible and practical to implement [21,22]. A few examples wherein LP methods were applied to improve the population group’s dietary pattern exist. In a community-based, clustered-randomized trial among Minangkabau women with dyslipidemia, the LP approach was used to formulate optimal food-based recommendations (FBRs). The consumption of promoted food (sea fish, soy protein, dark green leafy vegetables, and potato with skin) significantly increased and led to increased intake of problem nutrients (*n*-6 fatty acid, zinc, iron, and fiber) [23].

The LP approach in this study will be applied to the worker dietary pattern to identify the problem nutrient, find the optimal combination of daily foods reflected as food-based recommendations (FBRs), and a nutrient-dense canteen menu to improve the dietary quality of oil and gas workers who are predominantly overweight and have dyslipidemia. The nutrients of interest to be optimized were *n*-3 and *n*-6 polyunsaturated fatty acids (PUFAs), Eicosapentaenoic acid (EPA), Docosahexaenoic acid (DHA), and dietary fiber, while at the same time controlling the intake of sodium, total fat, and saturated fat within the acceptable levels.

## 2. Materials and Methods

### 2.1. Study Design

Using a cross-sectional study design, a dietary survey using a 1-day weighed food record, 24 h dietary recall, and 5-day food tally were conducted at the oil and gas worksite between February and March 2020. The cost of food per 100 g edible portion was obtained from the worksite canteen procurement.

This study was conducted in East Kalimantan Province, Indonesia. The province was chosen as it recorded the highest availability of oil and gas worksites. There were 12 sites located in East Kalimantan from a total of 82 sites across the country [24]. Recruitment of the site was based on the following criteria: (1) Recommendation from the Special Task Force for Upstream Oil and Gas Business Activities Republic of Indonesia (SKK Migas); (2) the company’s willingness to support this research.

The site of oil and gas was generally divided into two types based on the geographical location, which could be onshore (land-based) or offshore (platform located at the sea) [25]. Compared to their onshore counterparts, offshore locations imply an extreme physical environment in the middle of the sea, confined working and living quarters, lack of privacy, separation from family and local communities, perceived hazards of offshore work, and helicopter travels, contributing as stressors for workers’ physical and mental health. However, on their 12–14 days of working days, both offshore and onshore workers are facing heavy workloads with long working hours (12 h) [10]. In this study, one offshore and one onshore site were selected with the expectation that different workplace settings would allow us to assess a greater range of dietary patterns and a variety of food items so that the developed FBR would be applicable to oil and gas workers in both settings.

Food was provided by the company in the worksite canteen in a free-flow buffet style. This covers the mealtime of breakfast, lunch, dinner, and coffee breaks for the offshore site, but only breakfast on the onshore site. Lunch and dinner on the onshore site were pre-ordered with three choices: (1) traditional Indonesian cuisine, (2) western, and (3) plant-based menu. Supper could be requested for night-shift workers at both sites. The worker was restricted from eating any food outside the canteen; however, since the onshore site was surrounded by residential areas, they could easily go out to eat in nearby food stalls or make a delivery order, unlike the isolated offshore setting.

### 2.2. Study Participants

Participants were male oil and gas employees aged 30–49 years old with at least one year duration of employment and were working on shift for at least one week before the 7-day dietary survey. Workers diagnosed with a history of cardiovascular and/or chronic kidney diseases were excluded.

A list of all workers was obtained from each company’s health clinic. A total of 104 workers were eligible for this study, 52 workers were on shift, and from those 17 workers stayed less than seven days during data collection periods. In total, 35 oil and gas workers were recruited. Four respondents were excluded from the analysis due to incomplete record days (2) and under-reporting (2). Finally, 31 subjects were included in the analysis, consisting of 13 offshore workers and 18 onshore workers.

### 2.3. Data Collection

#### 2.3.1. Socio-Demographic Characteristics and Anthropometry

Information on socio-demographic characteristics, sleep duration, and smoking habits was collected by the enumerators through face-to-face interviews. Participants were asked to record their activities using Bouchard physical activity records on two non-consecutive working days and one day during the weekend. The activity was recorded based on the intensity category with a score in the range of 1–9, with a higher score indicating a heavier intensity. The metabolic equivalent (MET) values were calculated, and average MET values were used to estimate physical activity level (PAL) [26]. The results of biochemical assessment including blood glucose, total cholesterol, HDL-cholesterol, and LDL-cholesterol were obtained from workers’ annual medical check-up records from the health clinic.

Two repeated measurements of body weight, body height, waist circumference, and systolic and diastolic blood pressure were measured, and the average value was used in the analysis. The body weight was measured using the SECA 876 weighing scale and recorded to the nearest 0.1 kg. SECA 206 mechanical measuring tape was used to measure the height to the nearest 0.1 cm. Waist circumference was measured using a SECA 201 measuring tape to the nearest 0.1 cm. Systolic and diastolic blood pressure were measured using a digital sphygmomanometer. All measurements were taken by trained field enumerators with the standard procedure following the protocol of the WHO STEPwise approach to non-communicable disease risk factor surveillance [27].

#### 2.3.2. Dietary Assessment

Weekly food consumption was assessed using 1 day 24 h dietary recall (24HR), 1 day weighed food record (WFR), and 5 day food tally. The assessment of 24HR was conducted on the first day of data collection, the multiple-pass approach was chosen to reduce the recall bias [28,29]. Respondents were shown food pictures to help them estimate the portion size of foods consumed [30]. Food portions for weighed food record (WFR) were weighed using the Tanita digital scale for kitchen use model KD-160 with the precision ±1 g and were recorded by a trained enumerator. During mealtime in the canteen, the enumerator followed the study participants to weigh the food and beverages consumed. Additional food, snacks, and leftovers were also weighed.

The details of food preparation were observed directly at the company’s kitchen, and documentation of food recipes was recorded and confirmed with the head chef and/or nutritionist. The salt intake was recorded together with WFR, including (1) the weight of salt and sauces added at the cooking steps and salt added by the worker at the table, (2) salt from processed food obtained from the food label, and (3) in case of eating outside the canteens, salt reference value from Indonesian Ministry of Health was used to calculate the estimated salt absorption for all condiments [31,32]. Data for the 5-day food tally were obtained using a self-administered food record questionnaire to estimate the weekly frequency of each food item, which was verified every two days by enumerators to ensure completeness.

#### 2.3.3. Food Price Data

Information on the food availability and cost was obtained from the document of delivery order of the worksite canteen to the local supplier. The price was averaged between onshore and offshore companies. Data are expressed as cost (in Indonesian Rupiah, IDR) per 100 g edible portion.

#### 2.3.4. Food Composition Table

Food nutrient values were obtained from the Indonesian Food Composition Table (www.panganku.org) [33], except for the fatty acid contents which were borrowed from the United States Department of Agriculture Food Composition Table (https://fdc.nal.usda.gov/) [34] with water content adjustment [35].

#### 2.3.5. Recommended Energy and Nutrient Intake

Nutrient requirements were acquired from Indonesian Recommended Nutrient Intake (RNI) for males aged 30–49 years old [36]. The recommendation of fatty acid intake refers to the median of FAO-recommended dietary intakes for total fat and fatty acid in the adult population [37].

Energy requirement was calculated using the equations for estimating Basal Metabolic Rate (BMR) from body weight for males ages 30–49 years old available in the Optifood application multiplied by PAL. The physical activity level of active or moderately active was chosen given the occupational activities of oil and gas workers [38]. Ideal body weight (IBW) is determined as the target weight when the FBRs are followed. IBW was calculated by using body weight as a function of the actual population’s average height and target body mass index (BMI). Since the population has a high overweight and obesity prevalence, the upper limit of the normal BMI range (23.0 kg/m^2^) is used [39]. Based on the calculations, the ideal body weight was 69.5 kg.

### 2.4. Data Analysis

Data were entered using EpiData version 3.1. One week’s food consumption data (1 day 24 h dietary recall (24HR), 1 day weighed food record (WFR), and 5 day food tally) were prepared using the NutriSurvey program. Statistical analyses were completed with the IBM SPSS Statistic version 21. Data are presented as mean ± SD for normally distributed variables and the median (Q1–Q3) for non-normally distributed variables. Statistical significance was determined at a *p*-value < 0.05 level.

### 2.5. Linear Programming Applied to Formulate FBRs and Nutrient-Dense Menu

#### 2.5.1. Preparation of Linear Programming Model Parameters

Summary statistics from the one-week food consumption data (1 day 24 h dietary recall (24HR), 1 d weighed food record (WFR), and 5 day food tally), including a list of foods consumed, food serving sizes, and frequency of consumption, were used to define the linear programming model parameters. Food groups and sub-groups were classified based on the Optifood classification. The final LP model parameter consists of foods consumed by at least 5% of workers and nutrient-dense foods consumed by <5% of the worker but has the potential to be promoted for consumption. Food serving size was defined as the median serving size of each food item (g/meal). The minimum and maximum frequency were defined as the 5th and 95th percentile for each food item, food sub-group, and food group, respectively. All data were imported into the WHO Optifood Software (version 4.0.14.0).

#### 2.5.2. Development of Modelled Diets

All three Optifood modules were used during the LP analysis. Module I was operated to verify whether the solution was feasible to develop realistic diet models within the actual dietary data.

Module II (Draft recommendation) was used to identify the two best diets for achieving nutrient goals. This includes (1) one best diet within the population’s average food pattern (FP), and (2) one best diet with a larger range of food patterns that may deviate from average intake but still within the observed value, called no food pattern (NFP). Problem nutrients and potential nutrient-dense food items, food subgroups, and food groups to be promoted were identified through this module to help design alternative sets of FBRs. Problem nutrients were defined as nutrients that did not fully satisfy the RNI in the two best diets.

In Module III (Test recommendation), linear programming was used to compare alternative sets of FBRs for nutritional adequacy and cost. During the LP analysis, original slots for the 14 nutrients available in the software were modified with the inclusion of SFAs, PUFAs, omega 6, omega 3, EPA + DHA, fiber, and sodium. The nutrients that were replaced included iron, zinc, thiamine, riboflavin, niacin, vitamin B-6, and vitamin A RE, which were found to be adequate in this population of male oil and gas workers.

Two series of analyses were conducted using the Optifood software tool. In the first series of analyses, the actual portion size was used for the LP input model parameter. Considering that dietary fiber consumption was reported as very low in this population, the second series of analysis includes the prescribed portion.

The FBR which (1) had the highest %RNI worst-case scenario for protein, calcium, vitamin C, folate, vitamin B-12, vitamin A RAE, PUFAs, omega 6, omega 3, EPA + DHA, and fiber, (2) did not exceed ≥100% RNI of total fat, SFAs, and sodium, and (3) had the lowest cost was selected as the final FBR [40]. There were two methods of nutrient gap calculations: (1) best-diet (no-food pattern) minus 100% of RNI and (2) selected FBR minus 65% of RNI. The first approach was more suitable for the population that already has a similar pattern with FBRs. The second approach results in larger gaps; however, it is safer in terms of nutrient adequacy; therefore, this study used the second approach. Those gaps were inserted into the nutrient-dense snack menu from available ingredients in the company’s canteen food procurement list. Menu development was performed using NutriSurvey 2007 [41].

### 2.6. Ethical Approval

Ethical approval was obtained from the Ethical Committee of the Faculty of Medicine, Universitas Indonesia No 182/UN2.F1/ETIK/PPM.00.02/2020 issued on 20 February 2020. All participants signed the written informed consent.

## 3. Results

### 3.1. Socio-Demographic Characteristics and Anthropometry Status

Characteristics of the study groups are presented in Table 1. A total of 31 workers were included in the analysis, including 13 offshore workers and 18 onshore workers. Statistically significant differences in mean values between the groups were identified in working duration (years), blood pressure, and lipid profile.

The offshore worker reported a higher total cholesterol level (226.8 ± 38.1 mg/dL vs. 199.7 ± 25.7 mg/dL, *p* < 0.05) and diastolic blood pressure (83.7 ± 6.5 mmHg vs. 75.1 ± 8.4 mmHg, *p* < 0.01) as compared to the onshore counterparts. All the respondents had 12 h long working hours with a 14/14 schedule. Twenty workers (64.5%) were categorized as obese (BMI ≥ 25 kg/m^2^). The average body mass index (BMI) was 25.4 ± 3.2 kg/m^2^ and 15 workers (48.4%) had central obesity. Twenty-one of thirty-one workers did not have adequate sleep. The physical activity levels (PALs) of 48.4%, 25.8%, and 25.8% of the workers fell into the sedentary, moderate, and vigorous PALs, respectively.

### 3.2. Nutrient Intake

Table 2 shows the energy, macronutrients, fatty acids, and fiber intakes among the study participants. Actual energy, carbohydrate, and dietary fiber intakes were below the respective RNI for adult males. Proteins, fats, and carbohydrates contributed approximately 15.6%, 34.5%, and 50.7% of the total energy intake, respectively. PUFAs intake and the PUFAs:SFAs (P/S) ratio were lower than the recommended levels, while fiber intake was only 38.8% of the recommended levels. Saturated fat, cholesterol, and sodium exceeded the recommended intakes. As a percentage of daily energy intake, the offshore worker reported a higher contribution of protein (17.4% vs. 15.1%, *p* < 0.05) and saturated fat (15.9% vs. 14.2%, *p* < 0.05). Higher cholesterol intake was observed in the offshore group (621 g/day vs. 301 g/day, *p* < 0.01).

### 3.3. Dietary Pattern

Including foods consumed by at least 5% of the workers plus the nutrient-dense foods in each offshore and onshore group, 173 and 176 food items identified among each group, respectively. Combined food items between groups resulted in 201 food items, which consisted of 15 food groups and 55 food subgroups, and their weekly frequency of consumption was presented in Table 3. The main meals of the workers consisted of staple food, protein source food, and fruit/vegetables. Staple food includes refined and whole grain products, where refined grain (e.g., white rice) was the most frequently consumed staple food. Protein from animal-source foods (meat, fish, and eggs) was consumed most frequently compared to plant-sourced (legumes, nuts, and seeds) (median weekly frequency of 25 vs. 6, respectively). Vegetables were consumed frequently (median weekly frequency of 26) but in small portions (median of 18 g). Tomatoes and carrots were the most common vegetables consumed, with 94% and 90% participation rates, respectively.

Offshore workers consumed fruits less frequently (median: 9 vs. 20 times per week in offshore and onshore workers, respectively; *p* < 0.01). The most consumed fruits were watermelon and papaya (81% for each). Higher weekly consumption of processed meat was reported in the offshore group (median: 1 vs. 3 times per week in onshore and offshore workers, respectively; *p* < 0.01). Side dishes were cooked using palm oil, margarine, and coconut oil with a median frequency of added fat consumption 32 times per week (4–5 times daily). Salt was the most consumed food in the spices and herbs group with a frequency of 14–42 times/week (2–6 times daily). Salt was used as seasoning mainly when cooking animal products, legumes, and vegetables. Aside from mealtime, the respondents consumed snacks during break time. The snacks provided in the canteen consist of pancakes, pastries, cakes, fruits, and one-dish meals. Sweetened bakery products were consumed by 13–35% of the workers. The median weekly consumption of added sugar was 12 times per week with a median portion size of 7 g.

### 3.4. Problem Nutrients, Draft FBRs, and Nutrient-Dense Canteen Menu

Given the similar issues experienced by offshore and onshore oil and gas workers, including excess body weight, high intake of total fat, saturated fat, and sodium, as well as low intake of dietary fiber, the LP analysis was performed with combined food lists and food patterns. Table 4 shows the LP analysis results from Modules II and III. Five problem nutrients were identified in Optifood Module II (two best diets), namely calcium, folate, total PUFAs, *n*-6 PUFAs, and dietary fiber. Potential nutrient-dense food groups and food sub-groups that were identified in Module II and could be promoted to achieve dietary adequacy were vegetables, fish without bones, soybeans, and products such as poultry, fortified cereals, and whole grain products. Lettuce, carrot, soybean tempeh, mackerel fish, and chicken were identified as potential nutrient-dense food sources. These food subgroups and food items were used either separately or combined with other foods to establish the optimized FBR alternatives in Module III. Calcium and folate were considered as partial problem nutrients given that 100% of the RNI could not be achieved with the best diet (Module II) but could be achieved in the best-case scenario (Module III). Total PUFAs, *n*-6 PUFAs, and dietary fiber were categorized as absolute problem nutrients.

The second run on whole LP analysis was performed using the prescribed portion size. Using this scenario, dietary fiber was identified as a partial problem nutrient, and on the other hand, total PUFAs, *n*-6 PUFAs, and dietary fiber were categorized as absolute problem nutrients. This scheme did not increase the cost of the food and resulted in fewer problem nutrients compared to the actual portion size. This scheme was selected to be tested in Module III to identify the alternative sets of FBRs.

Table 5 shows the 14 alternative FBRs determined based on identified nutrient-dense foods and food groups or sub-groups and comparison on their worst-case scenario nutrient levels in Module III. Among these alternatives, the last FBR was the most promising, considering that it could demonstrate the highest RNIs percentage for all problem nutrients in the list. Box 1 shows the final FBR formulated to improve the intake of problem nutrients identified among oil and gas workers and the recommended minimum portion sizes for each food. Except for calcium, total PUFAs, *n*-6 PUFAs, and dietary fiber, the best diet developed by LP significantly increased %RNI from the baseline non-optimized diet. The gaps between 65% of RNI with minimized %RNI in the selected FBR were inserted into the nutrient-dense canteen snack menu. The list of the 14-day modified canteen snack menu, nutrient content, and cost are presented in Table 6.

Box 1 **Food-based recommendations formulated for male oil and gas workers.**

**Recommended messages of food-based recommendations**

1. Consume 3 main meals in a day consisting of grain foods, including:
  - At least 5 servings/week of whole grain products
2. Consume 2 servings/day of meat, fish, or poultry, including:
  - At least 8 servings/week of fish, including 4 servings/week of Mackerel
  - At least 5 servings/week of poultry
3. Consume 1–2 serving/day of legume, nuts, or seeds
4. Consume 2.5 servings/day of vegetables, including:
  - At least 7 servings/week of dark green leafy vegetable
5. Consume 1.5 servings/day of fruits, including:
  - At least 5 servings/week of vitamin C sources fruit
6. Limit fried food or foods cooked with margarine, butter, and coconut milk to a maximum of 3 servings/day
7. Limit the usage of added salt to a maximum of ½ teaspoon/day

**Recommended portion size/serving**

1 serving of brown rice milled = 100 g (cooked weight)
1 serving of fish = 75 g (wet weight)
1 serving of meat/poultry = 40 g (wet weight)
1 serving of tempeh = 50 g (cooked weight)
1 serving of peanut = 20 g (wet weight)
1 serving of vegetables = 100 g (wet weight)
1 serving of fruits = 80 g (wet weight)
1 serving of vegetable oil/butter/margarine = 5 g
1 serving of coconut milk = 30 g
½ teaspoon of salt = 1 g

## 4. Discussion

To the best of our knowledge, this study is the first to identify the problem nutrients and develop FBRs using the LP approach for a specific population of oil and gas workers. The LP analyses identified calcium, folate, total PUFAs, *n*-6 PUFAs, and dietary fiber as problem nutrients in the diet of oil and gas workers. Furthermore, we also addressed the remaining nutrient gaps by modifying the canteen snack menu. The findings from this preliminary study show that the combination of both FBRs and a modified canteen menu has great potential to ensure workers’ nutrient adequacy.

The present study observed a high prevalence of overweight and obesity among oil and gas workers (BMI ≥ 25 kg/m^2^), i.e., 64.5%. This was comparable to the previous studies in oil and gas workers in Indonesia and Italy, i.e., 49.5% and 62.4% [7,42]. Our study reports a high intake of saturated fat and cholesterol with a low intake of fiber. The median intake of total fat and saturated fat reported in this study was more than the tolerable limits, while the dietary fiber intake was only one-third of the recommended amount. Similar findings were also observed in oil and gas workers in Norway, Italy, and Australia [42,43,44]. Offshore workers consumed higher amounts of cholesterol, which was probably due to a higher intake of animal-source foods. Both onshore and offshore sites served double animal protein every meal (e.g., chicken and egg or meat and chicken); however, processed meat was never served at the onshore site, whereas it was still served at the offshore site. In addition, offshore workers tend to consume organ meat more frequently. Lack of fresh food was indicated as a major challenge at the offshore site due to the isolated setting [10].

The most common food groups consumed by oil and gas workers were added fats, grains and grain products, vegetable, and animal protein source foods (Median: 32, 28, 26, and 25 times per week). Some observed issues related to the food provision and cooking methods in the canteen were overuse of oil, salt, and sauces; most side dishes were prepared using deep-fried cooking, stir-fried with ketchup, cooked with coconut milk, or with butter and margarine. Snack was mainly cake and bakery which may contribute to the higher intake of saturated fat and sugar. We found only one study reporting dietary patterns of offshore workers in Norwegians which found that meat, vegetables, fresh fruits, seafood (shellfish), French fries, eggs, cream, and ice cream were important components of the diet of workers. The study highlighted that their diet pattern may contribute to the development of CVD among oil and gas workers [44].

The above findings strengthen the statement that, in workplaces where employees’ meals are provided by the company, a poor design of the meals may lead to negative consequences for their health. Food-based recommendations developed in this study can be beneficial to improve the food provision since they were developed from the actual weekly dietary patterns of the workers. LP analysis found that PUFAs, omega 6, and dietary fiber were absolute problem nutrients. These nutrients were known to be associated with dyslipidemia and metabolic syndrome (MetS) [45,46,47,48].

Using the LP approach, the final FBRs can achieve the recommended intake of vitamin C, omega 3, EPA + DHA, folate, vitamin B12, vitamin A, and omega 3 intakes for males aged 30–49 years old. Adherence to these FBRs will also ensure the intake of sodium and saturated fat within the recommended value. However, after increasing the intake of problem nutrients from about 20% RNI in the non-optimized diet to 50–70% RNI in the optimized diet, it was still below 65% RNI of PUFAs, omega 6, calcium, and dietary fiber (nutrient gaps). We therefore use these nutrient gaps to guide the modification of the canteen menu, i.e., the ingredients and the amounts. For example, although the fiber intakes of the workers were very low, some choices of whole grains were available in the canteen such as brown rice, oatmeal, and muesli. Therefore, the FBRs promoted the consumption of whole grain products a minimum of 5 times/week. This can increase the %RNI of dietary fiber from 9.2% to 23.5% in the worst-case scenario. Another example is shifting the type of fish to fatty fish as a source of *n*-3 PUFAs. The consumption of a minimum of eight servings per week of any type with half of it being fatty fish may contribute to ≥50% of RNI for *n*-3 PUFAs, EPA, and DHA (FBRs #1).

While previous LP studies suggested fortification and supplementation to fill the nutrient gaps (e.g., fortified infant cereal for under-two children, weekly iron supplementation for adolescent girls) [22,49], the current study intended to achieve the nutrient gap by modifying the canteen menu. The 14-day canteen snack menu was modified using the nutrient-dense food identified in the LP analyses. Contrary to the previous menu which was energy-dense foods (mainly cake and bakery), we proposed to reduce the food processing that uses butter and margarine as they contain high saturated fat. Nevertheless, to prevent rejection from drastic changes, we modified the ingredients using the identified nutrient-dense food from Module II LP analysis. For instance, the recipe for caramel pudding was modified into carrot pudding, brownies into oat cookies, and fried rissoles into nonfried spring rolls with vegetable filling. There were two coffee break times each day, the snack for the morning was modified cakes/bakeries and the second menu was legumes and vegetables or fruit as a source of PUFAs and dietary fiber. Consumption of these modified snacks can contribute up to 15–32%RNI of the problem nutrient. The cost of the snack was in the range of IDR 8000–12,000, and the cost of following the FBRs was IDR 46,732 (approximately USD 3), which was within the company’s budget. Adherence to the FBRs and consumption of the modified snacks can ensure the adequacy of 12 nutrients of interest.

To date, the LP analysis using Optifood software has been primarily used to address undernutrition problems in infants and young children or women of reproductive age and adolescent girls [22,49,50]. This study adds evidence on the LP modeling in the population in the workplace setting. This study also optimized the potential of the workplace setting by inserting the nutrient gaps into the canteen menu in addition to promoting the FBRs. A previous study amongst garment workers showed that, while lunch provision increased intakes of vitamin-A-rich fruits, it was not associated with higher dietary diversity scores. The authors suggest a more gap-oriented design of the lunch to improve the nutritional status of workers and the less consumed foods; this recommendation can be realized in our study by using the LP approach [51].

The main limitation of this study was its relatively small sample. Nevertheless, the respondents included in this study have similar characteristics to all workers based on the 2019 medical records (*n* = 104), which showed 77.4% overweight and obesity prevalence. Further intervention studies are needed to test the effectiveness of FBR promotion and nutrient-dense menu to reduce overweight/obesity and dyslipidemia amongst oil and gas workers.

## 5. Conclusions

The oil and gas workers reported poor dietary patterns including a high intake of total fat, saturated fat, and sodium, with a low intake of dietary fiber that may contribute to overweight and dyslipidemia. This study showed that a combination of FBRs and a modified canteen menu have great potential to optimize the diet of the workers. Further studies assessing the effectiveness of the developed FBRs and modified canteen menus to prevent overweight/obesity in workplace settings are needed.

## Figures and Tables

**Table 1:** Demographic and nutritional characteristics of oil and gas workers.

Variable	Total Workers, *n* = 31	Offshore, *n* = 13	Onshore, *n* = 18
Age (years)	39.5 ± 5.1	39.9 ± 5.9	39.3 ± 4.6
Working period (years)	7.0 (3.0–16.0)	3.0 (3.0–4.5)	12.5 (9.8–19) *
Body mass index (kg/m^2^)		25.7 ± 1.9	25.2 ± 3.9
Normal: 18.5–22.9	7 (22.6)	2	5
Overweight: 23.0–24.9	4 (12.9)	2	2
Obese: ≥25	20 (64.5)	9	11
Waist circumference (cm)		91.2 ± 4.7	88.1 ± 11.5
Central obesity: WC ≥ 90	15 (48.4)		
SBP (mmHg)		122.5 ± 11.5	118.7 ± 10.3
Hypertension: >140	1 (3.2)	1 (7.7)	0
DBP (mmHg)		83.7 ± 6.5 *	75.1 ± 8.4
Hypertension: >90	1 (3.2)	1 (7.7)	0
Lipid profile (mg/dL)			
LDL cholesterol		146.3 ± 52.9	135.1 ± 26.7
HDL cholesterol		61.9 ± 31.2	47.3 ± 7.4
Total cholesterol		226.8 ± 38.1 *	199.7 ± 25.7
Borderline-high: ≥200	19 (61.3)		
Fasting blood glucose (mg/dL)		89.3 ± 10.1	93.6 ± 12.7
Prediabetic: 100–125	5 (16.1)		
Inadequate sleep: <7 h/day	21 (67.7)	8	14
Ever-smoker	17 (54.8)		
Family history of NCDs	24 (77.4)		
Physical activity level	1.69 (1.64–2.05)	1.80 (1.60–2.19)	1.67 (1.63–1.84)
Sedentary: 1.40–1.69	15 (48.4)	5	10
Active/moderate: 1.70–1.99	8 (25.8)	3	5
Vigorous: 2.00–2.40	8 (25.8)	5	3

Abbreviations: DBP, Diastolic blood pressure; HDL, High-density lipoprotein; LDL, Low-density lipoprotein; NCDs, Non-communicable diseases; HDL, High-density lipoprotein; LDL, Low-density lipoprotein; SBP, Systolic blood pressure, WC, Waist Circumference. * Test result with the significantly higher mean difference between the offshore and onshore workers (*p* < 0.05).

**Table 2:** Actual energy and selected nutrient intakes ^†^ of oil and gas workers.

Nutrients	Unit	RNI ^‡^	Total (*n* = 31)		Offshore (*n* = 13)		Onshore (*n* = 18)	
			Median	Min–Max	Median	Min–Max	Median	Min–Max
Energy	(kcal)	2550	2313	2006–2599	2067	1771–2377	2438	2152–2709
Protein	(g)	65	84.0	75.6–99.4	84.0	76.8–101.1	85.5	75.3–100.7
	(%)	10.2	15.6	14.5–17.5	17.4 *	15.2–18.4	15.1	13.4–16.2
Total fat	(g)	76.5	89.8	64.6–112.2	81.6	64.0–112.9	93.4	75.0–106.1
	(%)	27	34.5	29.0–40.3	34.5	31.0–43.2	35.0	28.5–39.7
Total PUFAs	(g)	24.1	12.6	9.6–16.9	9.6	7.9–15.4	14.0	11.8–17.6
	(%)	8.5	5.1	3.9–6.2	4.5	3.5–6.1	5.6	4.5–6.8
*n*-6 PUFAs	(g)	17	3.88	2.43–6.33	5.12	3.05–6.35	2.98	1.71–5.61
*n*-3 PUFAs	(g)	1.6	0.83	0.64–1.05	0.83	0.59–1.02	0.81	0.67–1.06
EPA + DHA	(g)	1.125	0.370	0.150–0.495	0.175	0.140–0.370	0.428 *	0.321–0.565
MUFAs	(g)	4.3	29.0	24.1–37.0	28.6	24.3–37.4	30.0	21.0–36.7
	(%)	1.5	11.6	9.9–14.2	12.7	10.5–15.6	10.1	9.2–13.5
SFAs	(g)	<28.3	39.8	31.6–43.1	38.9	31.1–45.7	39.9	30.5–43.3
	(%)	10	14.6	12.9–18.0	15.9 *	14.5–19.1	14.2	11.8–15.9
PUFAs/SFAs	ratio		0.36	0.27–0.43	0.27	0.23–0.36	0.41 *	0.32–0.46
Cholesterol	(mg)	<300	460	225–690	621 *	462–841	301	218–503
Carbohydrates	(g)	415	270.8	218.2–333.2	247.3	205.7–275.1	313.4 *	243.7–358.9
	(%)		50.7	44.5–56.1	49.2	40.0–55.2	51.6	45.6–58.9
Dietary fiber	(g)	36	14.3	12.2–20.0	13.2	9.9–20.0	15.8	13.6–20.6
Sodium	(mg)	1500	3174	2740–3777	3174	2476–3736	3181	2776–3785

Abbreviations: EPA, Eicosapentaenoic acid; DHA, Docosahexaenoic acid; MUFAs, Monounsaturated fatty acids; PUFAs, Polyunsaturated fatty acids; RNI, Recommended Nutrient Intake; SFAs, Saturated fatty acids. ^†^ Intake was the average of 1-day 24 h dietary recall and 1-day weighed food record. ^‡^ RNI for energy and most nutrients refers to the Indonesian RNI while RNI for fat and fatty acids refers to Food and Agriculture Organization recommendations for fat and fatty acid intake (FAO, 2010). * Test result with the significantly higher mean rank difference between offshore and onshore workers (*p* < 0.05).

**Table 3:** Weekly frequency of food group and sub-group consumption (servings/week).

Food Group/ Food Subgroup	Median Serving Size	Time Consumed per Week						*p* Value ^a^
		Total (*n* = 31)		Offshore (*n* = 13)		Onshore (*n* = 18)		
		Median	P_5_–P_95_	Median	P_5_–P_95_	Median	P_5_–P_95_	
Added fats	6	32	19–48	29	18–42	35	22–50	0.123
Added sugars	8	12	3–22	10	5–17	15	5–22	0.166
Bakery and breakfast cereals	60	8	1–20	4	1–12	8	3–24	0.221
Beverages	13	6	0–19	6	0–19	4	1–13	0.421
Composites (mixed food group)	50	2	0–5	4	1–5	2	1–4	0.007 ^b^
Dairy products	53	4	0–13	5	1–13	5	2–13	0.505
Fruits	88	12	3–33	9	2–25	20	9–35	0.008 ^b^
Grains and grain products	40	28	8–40	27	8–35	29	9–42	0.422
Fortified grain and products	47	7	4–9	5	4–8	8	6–9	0.051
Refined grain products	63	20	1–32	25	9–34	29	5–43	0.968
Whole grain products	55	2	0–8	3	0–5	2	0–7	1.000
Legumes, nuts, and seeds	27	6	1–18	5	1–9	8	2–19	0.042 ^b^
Cooked beans and peas	40	0	0–1	0	0–0	0	0–2	0.078
Nuts, seeds, and unsweetened products	24	1	0–2	1	0–3	1	1–2	0.787
Soybeans and products	29	4	0–18	3	1–6	5	0–15	0.031 ^b^
Meat, fish, and eggs	50	25	17–33	26	18–34	23	17–32	0.166
Eggs	50	6	2–12	7	3–12	4	0–11	0.084
Fish without bones	54	6	2–10	5	1–8	6	1–11	0.129
Organ meat	25	0	0–2	0	0–2	0	0–1	0.024 ^b^
Poultry, rabbit	46	6	1–11	6	2–9	7	0–10	0.615
Processed meat	27	2	0–5	3	1–5	1	0–3	0.007 ^b^
Red meat	75	3	0–6	3	1–5	2	0–6	0.224
Seafood	22	0	0–1	1	0–3	1	0–3	0.966
Small, whole fish, with bones	33	0	0–1	0	0–1	0	0–1	0.536
Miscellaneous	1.5	72	47–123	65	47–91	87	50–125	0.017 ^b^
Condiments, herbs, spices	0.9	58	37–109	56	37–76	74	5–113	0.045 ^b^
Savory spreads and sauces	3	11	4–7	9	4–15	12	0–16	0.015 ^b^
Sweet sauce and jams	12	1	0–6	1	0–5	1	0–7	0.525
Savory snacks	25	1	0–5	2	0–5	3	1–9	0.627
Starchy roots and plants	30	2	0–5	2	1–5	2	1–4	0.008 ^b^
Sweetened snacks and desserts	54	1	0–5	1	0–7	3	1–5	0.077
Vegetables	18	26	10–47	24	11–46	29	11–46	0.471

^a^ Significance was tested for a mean rank difference between offshore and onshore worker’s food frequency. ^b^ Mann–Whitney U test with significance value *p* < 0.05.

**Table 4:** Comparison of nutrient levels and minimized costs of the two best diets (module II), worst-case and best-case scenario diets (module III) using actual portion size and prescribed portion size.

Analysis	Achievement of Nutrients (%RNI)														
	Protein	Fat	Ca	Vit C	Sodium	SFAs	PUFAs	EPA + DHA	Folate	Vit B12	ω − 6	Vit A	Fiber	ω − 3	Cost IDR/d
Actual Portion Size															
Best diet (food patterns)	186.6	136.7	75.0	105.2	151.3	172.0	60.9	98.0	78.0	307.6	60.4	117.1	41.9	126.1	74,098
Best diet (no-food pattern)	188.4	139.2	**86.3 ^a^**	130.5	214.6	169.3	**67.2 ^a^**	100.0	**92.4 ^a^**	279.5	**69.1 ^a^**	163.2	**60.4 ^a^**	128.1	81,653
Best-case scenario	212.7	158.8	101.2	293.5	266.5	209.9	**74.4 ^b^**	109.4	109.0	371.2	**75.5 ^b^**	258.1	**78.0 ^b^**	140.6	95,796
Worst-case scenario	118.3	102.1	**26.5 ^c^**	**4.4 ^c^**	67.2	108.6	**35.6 ^c^**	11.5	**22.3 ^c^**	**60.6 ^c^**	**20.9 ^c^**	**43.6 ^c^**	**14.4 ^c^**	**42.1 ^c^**	41,589
Prescribed Portion Size															
Best diet (food patterns)	170.3	132.1	86.7	194.8	123.2	153.4	62.3	100.0	100.0	221.2	60.3	230.6	57.2	126.6	59,399
Best diet (no-food pattern)	195.0	129.5	100.0	345.1	157.4	133.8	**76.9 ^a^**	109.4	128.9	207.6	**64.3 ^a^**	299.0	**98.0 ^a^**	136.5	73,820
Best-case scenario	209.3	150.9	143.5	545	237.1	189.7	**80.2 ^b^**	124.2	180.1	326.4	**81.5 ^b^**	403.5	111.4	169.3	96,681
Worst-case scenario	113.0	95.3	**27.5 ^c^**	**17.3 ^c^**	**55.8 ^c^**	89.2	**31 ^c^**	**11.2 ^c^**	**28.2 ^c^**	**42.7 ^c^**	**14.7 ^c^**	**40.8 ^c^**	**23.5 ^c^**	**29.4 ^c^**	37,531

Abbreviations: EPA, Eicosapentaenoic acid; DHA, Docosahexaenoic acid; FBRs, food-based recommendations; MUFAs, Monounsaturated fatty acids; PUFAs, Polyunsaturated fatty acids; RNI, Recommended Nutrient Intake; SFAs, Saturated fatty acids; Vit, Vitamin. ^a^ Bold in Best diet (no-food pattern) refers to a problem nutrient, i.e., model achievement could not meet 100% RNI. ^b^ Bold in Best-case scenario refers to absolute problem nutrient, i.e., maximized scenario could not meet 100% RNI. ^c^ Bold in Worst-case scenario refers to dietary inadequacy, i.e., minimized scenario could not meet 65% RNI.

**Table 5:** Comparison of nutrient levels and minimized costs of the two best diets (module II), worst-case and best-case scenario diets (module III), and alternative sets of FBRs tested (module III: worst-case scenario only) using prescribed portion sizes.

Analysis	Achievement of Nutrients (%RNI)														
	Protein	Fat	Ca	Vit C	Sodium	SFAs	PUFAs	EPA + DHA	Folate	Vit B12	ω − 6	Vit A	Fiber	ω − 3	Price (IDR)
Best diet (food patterns)	204.9	113.8	149.2	116.3	143.6	127.0	61.2	100.0	90.9	329.0	64.5	106.8	43.9	124.2	72,086
Best diet (no-food pattern)	207.3	125.3	127.3	134.3	201.1	149.8	**69.2 ^a^**	100.0	100.0	310.2	**71.2 ^a^**	144.4	**63.7 ^a^**	128.4	84,057
Best-case scenario without FBRs	247.6	158.8	191.5	308.7	284.2	209.9	**74.4 ^b^**	110.3	120.7	440.4	**75.6 ^b^**	260.6	**79.4 ^b^**	141.9	100,890
Worst-case scenario without FBRs	118.3	79.5	**26.5 ^c^**	**4.4 ^c^**	**64.1 ^c^**	76.8	**26.0 ^c^**	**9.2 ^c^**	**22.3 ^c^**	**60.6 ^c^**	**11.7 ^c^**	**24.6 ^c^**	**9.2 ^c^**	**28.5 ^c^**	39,691
Alternative sets of FBRs tested (worst-case scenarios only)															
WholeGrain5	113.0	95.3	27.5	17.3	55.8	89.2	31.0	11.2	28.2	42.7	14.7	40.8	23.5	29.4	37,531
LNS7, SoybeanP5	126.1	99.3	34.5	17.7	56.9	91.3	37.6	12.1	33.6	48.2	15.5	46.6	25.1	30.6	37,894
LNS10	130.0	100.8	35.8	18.2	57.6	93.0	40.0	12.5	36.4	51.0	16.3	49	26.3	31.0	38,016
MFP21, Fish8, Mackerel4	123.8	95.6	28.7	18.3	56.5	89.2	32.1	67.2	28.2	77.6	14.7	42.1	23.6	68.9	37,779
MFP14, Fish8, Mackerel4	123.8	95.6	28.7	18.3	56.5	89.2	32.1	67.2	28.2	77.6	14.7	42.1	23.6	68.9	37,779
Vegetable18, DGLV7	113.0	95.3	30.9	33.8	55.8	89.2	31.0	11.6	35.0	42.7	14.7	72.8	27.3	29.8	37,789
Fruit11, VitCSourcesFruit5	124.1	102.3	35.2	27.8	65.0	98.9	34.8	14.1	38.3	61.1	19.8	55.3	31.7	35.6	42,636
Poultry5, NutSeedandProduct2	115.1	98.0	27.9	17.3	55.8	89.8	34.9	11.3	31.3	44.2	23.5	42.2	24.9	29.6	37,531
AddedFat21	113.0	95.4	27.5	17.3	55.8	89.3	31.0	11.2	28.2	42.7	14.7	40.8	23.5	29.4	37,531
Salt7	113.0	95.3	27.5	17.3	55.8	89.2	31.0	11.2	28.2	42.7	14.7	40.8	23.5	29.4	37,531
FBRs1	135.9	99.4	35.7	18.6	57.5	91.3	38.6	68.0	33.8	83.0	15.5	47.9	25.2	70.0	38,111
FBRs2	151.4	109.5	51.9	67.3	78.9	106.5	45.8	71.9	59.4	105.5	24.4	100.5	49.1	79.9	44,889
FBRs3	157.1	114.4	55.9	94.4	86.9	111.7	51.1	73.0	70.3	112.7	33.1	110.0	57.8	83.7	46,733
Final FBRs *	157.9	112.5	56.0	94.8	86.9	111.7	49.8	73.0	69.4	112.7	28.2	110.0	57.8	83.7	46,732

Abbreviations: EPA, Eicosapentaenoic acid; DGLV, dark green leafy vegetables; DHA, Docosahexaenoic acid; FBRs, food-based recommendations; LNS, Legumes Nuts Seeds; MFP, Meat Fish Poultry; MUFAs, Monounsaturated fatty acids; PUFAs, Polyunsaturated fatty acids; RNI, Recommended Nutrient Intake; SFAs, Saturated fatty acids; Vit, Vitamin. ^a^ Bold in Best diet (no-food pattern) refers to a problem nutrient, i.e., model achievement could not meet 100% RNI. ^b^ Bold in Best-case scenario refers to absolute problem nutrient, i.e., maximized scenario could not meet 100% RNI. ^c^ Bold in Worst-case scenario refers to dietary inadequacy, i.e., minimized scenario could not meet 65% RNI. FBRs 1: WholeGrain5, LNS7, SoybeanProduct5, MFP21, Fish8, Mackerel4; FBRs 2: Vegetable18, DGLV7, Fruit11, VitCSourcesFruit5, WholeGrain5, LNS7, SoybeanProduct5, MFP21, Fish8, Mackerel4; FBRs 3: Poultry5, NutSeedandProduct2, AddedFat21, Salt7, Vegetable18, DGLV7, Fruit11, VitCSourcesFruit5, WholeGrain5, FortifiedCereal2, LNS7, SoybeanProduct5, MFP21, Fish8, Mackerel4; FBRs4 *: WholeGrain5, MFP14, Fish8, Mackerel4, LNS10, Vegetable18, DGLV7, Fruit11, VitCSourcesFruit5, AddedFat21, Salt7. * selected FBR.

**Table 6:** Nutrient content of original and modified snacks provided at the worksite canteen.

Day	Original Snack (O)	Modified Snack (M)	Energy (kcal)		PUFAs (g)		*n*-6 PUFAs (g)		Dietary Fiber (g)		Ca (mg)		Price in K (IDR)	
			O	M	O	M	O	M	O	M	O	M	O	M
1	Vegetable fritter	*Karedok*	383	409	1	4.9	1	4.4	1	6.1	70.2	108	4	12
	Brownies	Oat cookies												
2	Caramel pudding	Carrot pudding	343	393	1	5.7	1	5.6	0	6.6	50.9	193	8	11
	*Kue lumpur*	Boiled peanut and orange												
3	Croissant	Pao with tuna	413	403	1	6.3	1	5.3	1	13	34.9	159	8	9
	Putu ayu	Boiled soybean and guava												
4	Rissoles	*Lumpia basah* with vegetable filling	291	393	1	9.2	1	9	0	5.1	51	56.2	2	10
	Sponge white	Roasted sunflower seeds and banana												
5	Sausages Pizza	Wheat bread with peanut butter	390	428	1	5.6	1	5.5	0	5.3	58.4	171	6	10
	Muffin	Sweet potato muffin												
6	Pastel	Fish fillet with vegetable filling	389	406	1	6.3	1	5.3	0	6.9	58	134	3	12
	Chocolate ganache	Low-fat yogurt with muesli												
7	Fried fries	Potato cream soup with green peas	420	384	2	5.3	1	4.8	0	9	113	143	4	10
	Cream puff	Corn and pineapple salad												
8	Vanilla pudding	Steamed tofu	282	350	1	5.7	1	5.4	0	3.6	62.2	216	28	11
	Onion bread	Cucumber salad with olive oil dressing												
9	Bitter Ballen	A chicken ball with tomato sauce	350	335	2	6	1	5.5	1	3.3	113	67.1	4	9
	Danish pastry	Yunani salad												
10	Steamed sponge cake	Cassava steamed sponge cake	375	424	1	5.2	1	5	0	4.1	45.5	134	4	9
	Coffee bun	Bangkok salad												
11	Fried banana with chocolate	Homemade banana cereal bar	391	415	2	5.8	0	5.3	2	8.1	31.5	203	15	11
	Marble cake	Vegetable salad with cornflakes												
12	Éclair	Siomay tofu	248	344	1	6.1	1	5.8	0	2.7	25.7	190	11	10
	Rainbow cake	Cabbage salad with lemon-kiwi dressing												
13	Chicken puff pastry	Thousand island chicken soup	343	341	1	5.3	1	4.9	0	4.8	16.2	235	7	10
	Strawberry flavored pudding	Fruit salad												
14	Pancake	*Lontho* peanut cake	440	359	2	5.2	1	4.8	0	3.2	93.9	123	6	8
	Chicken Pizza	Caesar salad with grilled chicken												
Average nutrient content			361	385	1	5.9	1	5.5	1	5.8	58.8	152	6	10
%RNI			13	14	5	22	4	32	1	16	5.9	15.2	-	-

## Data Availability

The data supporting the reported results are available upon request from the corresponding author.
